# Curcumin Attenuation of Lipopolysaccharide Induced Cardiac Hypertrophy in Rodents

**DOI:** 10.1155/2013/539305

**Published:** 2013-10-21

**Authors:** Rupak Chowdhury, Ramadevi Nimmanapalli, Thomas Graham, Gopal Reddy

**Affiliations:** ^1^College of Veterinary Medicine, Nursing and Allied Health, Tuskegee University, Tuskegee, AL 36088, USA; ^2^Philadelphia College of Osteopathic Medicine, School of Pharmacy, Suwanee, GA, USA

## Abstract

To study the ameliorating effects of curcumin in lipopolysaccharide (LPS) induced cardiac hypertrophy, mice were assigned to 4 groups (3 males and 3 females in each group): (A) control, (B) curcumin: 100 **μ**g/kg of body weight by intraperitoneal route (IP), (C) LPS: 60 mg/kg (IP), and (D) LPS + curcumin: both at previously stated concentrations by IP route. All mice were sacrificed as 12 hr and 24 hrs groups accordingly after LPS injection. The hearts were collected, photographed for cardiomegaly, and weighed to compare heart weight/brain weight (HW/BW) in mg/mg. For immunohistochemistry, the tissue sections were exposed to histone H3, H4 and acetylated histone H3, H4 antibody. LPS induced a significant increase in histone acetylation as shown by intense staining. In curcumin + LPS treated mice nuclear staining was similar to the control group indicating that curcumin traversed the histone acetylation activity of the LPS. To further check the mechanism of action of curcumin, p300 protein acetylation levels were analyzed. This study suggests that the probable mechanism of action of curcumin is via the reduction of p300 HAT activity.

## 1. Introduction

 In the United States, foodborne pathogens cause 76 million illnesses, 325,000 hospitalizations, and 5,000 deaths annually [1]. Some of the main pathogens behind these sicknesses are gram-negative bacteria such as *Salmonella, Escherichia*, and *Campylobacter* [2]. Infections with gram-negative bacteria may lead to endotoxemia caused by lipopolysaccharides (LPS) which is a complex of glycolipids made up of two distinct regions. These are hydrophilic polysaccharide region (composed of O antigen and core oligosaccharide) and hydrophobic regions known as lipid A [3]. Lipid A is responsible for most of the LPS induced biological effects. Since lipid A is the innermost component of LPS, it is closely adherent to the inner cell wall of bacteria and is generally not released (and therefore not toxic) until the death of the bacterial cell [4]. LPS is heat stable and not strongly immunogenic, so it cannot be converted to a toxoid [4]. LPS increases histone acetylation in hypertrophic myocardium. Histone modification is the central point for the control of cardiac growth and gene expression in response to acute and chronic stress stimuli [5]. In the prevention and treatment of cardiac disease, histone modification and the signaling pathways manipulation is a major therapeutic step [5]. Histone acetyltransferases (HATs) mediate acetylation of histone tails, loosening the interaction between DNA and histones. Acetylation of histones reduces their overall positive charge, thus decreasing their tight interactions with negatively charged DNA [5]. Thus, it activates the transcriptional pathway and gene expression [6]. Histone deacetylases (HDACs) can remove the acetyl groups on amino-terminus of histones, restore tight interactions between DNA and histones, and deactivate the transcriptional pathway [5]. Therefore, HATs (histone acetyltransferases) and HDACs (histone deacetylases) activity determines the transcriptional activation or repression. 

HAT activity in cardiac muscle is determined by p300 that causes modification of chromatin and associated transcription factors and gene expression [7]. Studies done by Yanazume et al., 2003, showed that agonist-induced cardiac hypertrophy accentuates p300 transcriptional activity and that this agonist-mediated cardiac hypertrophy is reversed by the blocking of p300-HAT activity [8]. Therefore, p300-HAT is a tempting target to treat or prevent myocardial hypertrophy. Curcumin is an inhibitor of p300-HAT but very little is known about whether this regulatory effect is related to a protective role in cardiac dysfunction [9].

Curcumin which is derived from turmeric (*Curcuma longa* plant) is a tropical plant that is native to Southern and Southeastern tropical Asia [10]. It is a perennial herb within the ginger family and is known for its yellow-orange color and for numerous therapeutic applications [10]. Curcumin makes up to 2–5% of the spice turmeric. [10]. Curcumin is a polyphenolic compound and has been used in the treatment of many conditions, including cardiovascular diseases [9]. 

This study was designed to determine the potential for curcumin to attenuate LPS induced cardiac hypertrophy in rodents. Additionally, the mechanism of action for this attenuation was investigated. 

## 2. Methods

### 2.1. Materials

Curcumin C3 complex (R) was a kind gift from Sabinsa Corporation, Hyderabad, India. The patented C3 complex (R) contains curcumin and its derivates demethoxycurcumin and bisdemethoxycurcumin, also known as curcuminoids. Curcumin is 1–5% of the turmeric. LPS sc-3535 was obtained from Santa Cruz Biotechnology as a white to yellow lyophilized powder. For the live and dead experiment, LPS was used as 1.5 mg/mice (dissolved in distilled water), and in curcumin attenuation of LPS induced cardiac hypertrophy, LPS was used as 60 mg/kg body weight dose (dissolved in distilled water). Histone H3 antibody was obtained from Santa Cruz Biotechnology, Santa Cruz, CA. It is a purified antibody. Histone H3 (N-20), was produced against a peptide mapping at the N-terminus of histone H3 of human origin. Histone H4 was obtained from Santa Cruz Biotechnology, Santa Cruz, CA. histone H4 (N-18), purified goat polyclonal antibody, was produced against a peptide mapping at the N-terminus of histone H4 of human origin. p300 (C-20) was obtained from Santa Cruz Biotechnology, Santa Cruz, CA. p300 (C-20), affinity purified rabbit polyclonal antibody, was raised against a peptide mapping at the C-terminus of p300 of human origin. Acetyl-histone H3 (Lys9) antibody was obtained from cell signaling Technology, Danvers, MA. Endogenous levels of Histone H3 were detected by acetyl-histone H3 (Lys9) antibody only when acetylated at lysine 9. Acetyl-histone H4 (Lys12) antibody was obtained from cell signaling Technology, Danvers, MA. Endogenous levels of histone H4 were detected by acetyl-histone H4 (Lys12) antibody only when acetylated at Lys12. 

Acetyl-CBP (Lys1535)/p300 (Lys1499) antibody was obtained from Cell Signaling Technology, Danvers, MA. Acetyl-CBP (Lys1535)/p300 (Lys1499) antibody detects endogenous levels of CBP or p300 only when acetylated at lysine 1535 or lysine 1499, respectively. 

### 2.2. Animal Models

The Animal Care and Use Committee of Tuskegee University approved the experimental protocol (protocol number R0804-12-1). Eight breeding pairs of mice (*Mus musculus*) strain 129S6/SvEeV, IL-10^−/−^, were received from the Gnotobiotic Unit of The Mutant Mouse Resource Center, University of North Carolina, Chapel Hill, NC (IACUC approval number R076-10-2). The mice were housed under specific pathogen-free (SPF) conditions in the Comparative Medicine Resource Center (CMRC), Tuskegee University, and bred via brother : sister mating to produce the mice used in this study. Subsequently, 48 of the resulting mice were randomly assigned to one of four treatment groups. The adult 129SvEv mice (6–8 weeks old) used in the current study were assigned to 4 groups (3 males and 3 females in each group): (A) control, (B) curcumin: 100 *μ*g/kg of body weight, (C) LPS: 60 mg/kg, and (D) LPS (60 mg/kg) + curcumin (100 *μ*g/kg of body weight). Experiments were run for 12 hrs and 24 hrs. Then, following a 24 acclimation period, curcumin and Curcumin + LPS treated groups received intraperitoneal (i.p.) injections of curcumin (100 *μ*g/kg of body weight). The curcumin suspension was prepared using an olive oil solution. One day after the pretreatment with 100 *μ*g/kg curcumin, the LPS and curcumin + LPS groups received LPS: 60 mg/kg i.p. injection of LPS. All mice in the 12 hrs subgroups were sacrificed with CO2 12 hrs after LPS injection. All mice in the 24 hrs subgroups were sacrificed with CO2, 24 hrs after LPS injection.

 The hearts were collected and photographed for cardiomegaly. Hearts and lungs of the sacrificed mice were dissected and weighed to compare heart weight/brain weight (mg/g) and lung weight/brain weight (mg/g) ratios in LPS and curcumin treated mice. The collected organs were then preserved in 10% regular formalin in 50 mL tubes.

### 2.3. Immunohistochemistry

Immunohistochemistry is the process of detecting antigens in cells of a tissue section by exploiting the principle of antigen-antibody binding specificity. The antibody is conjugated to peroxidase that catalyses a color-producing reaction. The sections of murine tissue were placed on the heated water bath and picked up, placed on the slides, and placed in a rack. After all slides had been cut and placed in a rack they were put into a 60 degrees Celsius oven over night. The slides were deparaffinized by going through 3 changes of xylene 2 minutes each, followed by 3 changes of 100% alcohol at 1 minute and one change of 95% alcohol at 1 minute. Slides were washed in running tap water for 1 minute and stored in distilled water slides. The slides were then subjected to antibody pretreatment.

### 2.4. Statistical Analysis

Comparisons between 2 groups were performed by paired Student's *t*-test. Each group was normally distributed, and the variances were equal in the two groups. The test statistic, *t*_*s*_, was calculated using a formula that has the difference between the means in the numerator. The denominator was the standard error of the difference in the means, which got smaller as the sample variances decrease or the sample sizes increase. Thus *t*_*s*_ got larger as the means got farther apart, the variances got smaller, or the sample sizes increased.

A *P* value less than 0.05 was considered significant.

## 3. Results and Discussion

### 3.1. Curcumin Protects Mice against LPS Induced Cardiomegaly

**Figure 1:**
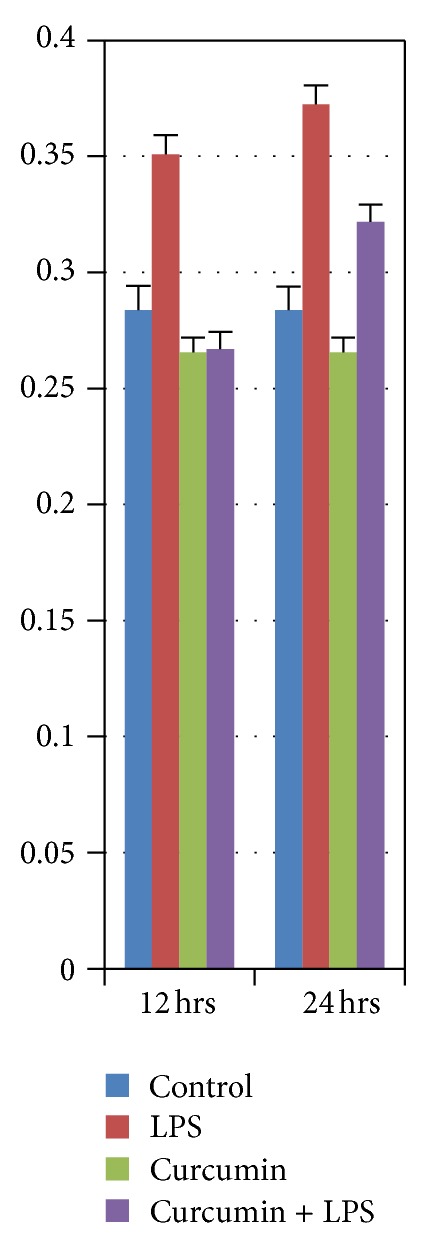
Heart wt./brain wt. ratios of rats at 12 hrs and 24 hrs after respective treatment. The LPS treated group was significantly different from all other groups (*P* < 0.04).

**Figure 2:**
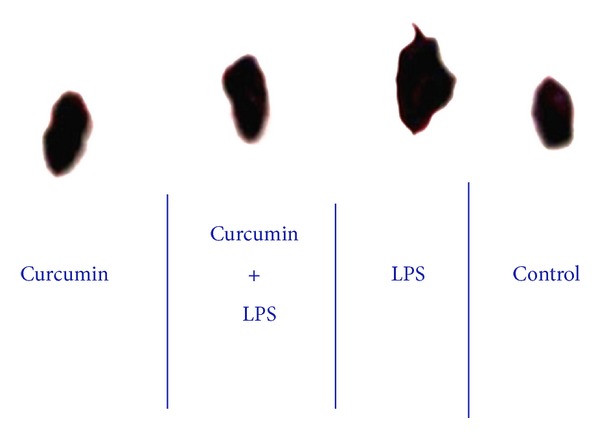
Curcumin protects mice against LPS induced cardiomegaly. The heart collected 24 hrs after LPS treated mice group showed significant cardiomegaly, and the heart from the LPS + Curcumin treated group showed that curcumin reverses LPS induced cardiomegaly.

Mice in the LPS treated group showed significant cardiac hypertrophy as measured by heart weight/brain weight (HW/BW) ratio.  Figure 1 illustrates that the LPS treated group showed significant cardiomegaly. In 12 hrs subgroup, the *P* = .04 denotes a significant difference between the control and LPS, and in the 24 hrs subgroup, the *P* = .0026 denotes a significant difference between the control and LPS treated groups. Curcumin treatment inhibited LPS induced cardiomegaly. Mice, in the LPS + curcumin treated group, showed attenuation of cardiac hypertrophy as measured by heart weight/brain weight (HW/BW) ratio (Figure 1). In 12 hrs subgroup, the *P* = .005 denotes a significant difference between the LPS and LPS + curcumin, and in 24 hrs subgroup, the *P* = .04 denotes a significant difference between the LPS and LPS + curcumin treated groups. No significant changes were observed in the mice treated with either curcumin or control group (Figure 1). In Figure 2, the heart collected from LPS treated mice group showed significant cardiomegaly. Figure 2 also shows that curcumin inhibited LPS induced cardiomegaly in our murine model.

### 3.2. Curcumin Attenuates Histone H3 Acetylation

Chromatin structure modulation has critical role in the regulation of transcription in eukaryotes. DNA wound around eight core histone proteins (two each of H2A, H2B, H3, and H4) form the nucleosome which is the primary building block of chromatin [11]. The amino-terminal tails of core histones pass through various posttranslational modifications, including acetylation, phosphorylation, methylation, and ubiquitination. These modifications happen in response to various stimuli and serve to open the transcriptional machinery, increase the accessibility of chromatin to transcription factors, and ultimately result gene expression [12]. Acetylation of H3 has a central role in histone deposition and chromatin assembly in some organisms. Histone acetylation is associated with pathological cardiac hypertrophy and heart failure [13]. HATs acetylate histone proteins, relax chromatin, and expose prohypertrophic genes for activation by cardiogenic transcription factors. Inhibition of histone acetylation has a central role for the antihypertrophic activity of curcumin [9].

**Figure 3:**
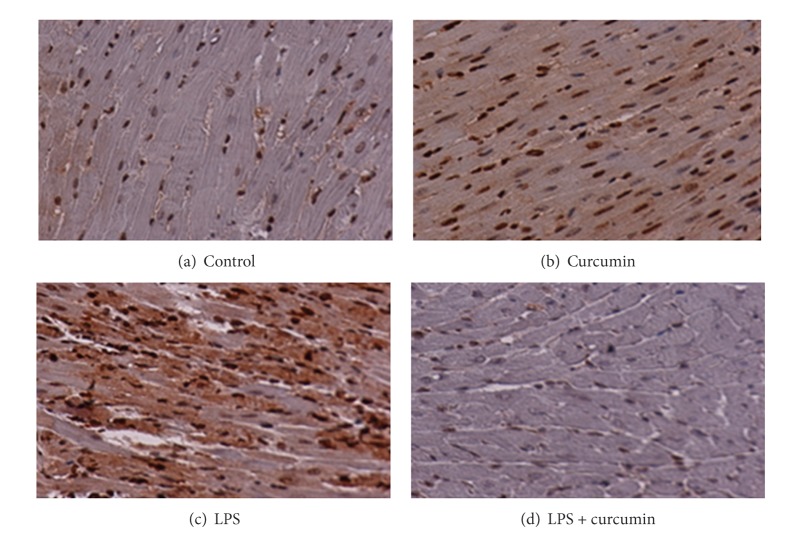
Histone proteins were detected by histone H3 antibody in control (a), curcumin (b), LPS (c), and curcumin + LPS (d) groups.

**Figure 4:**
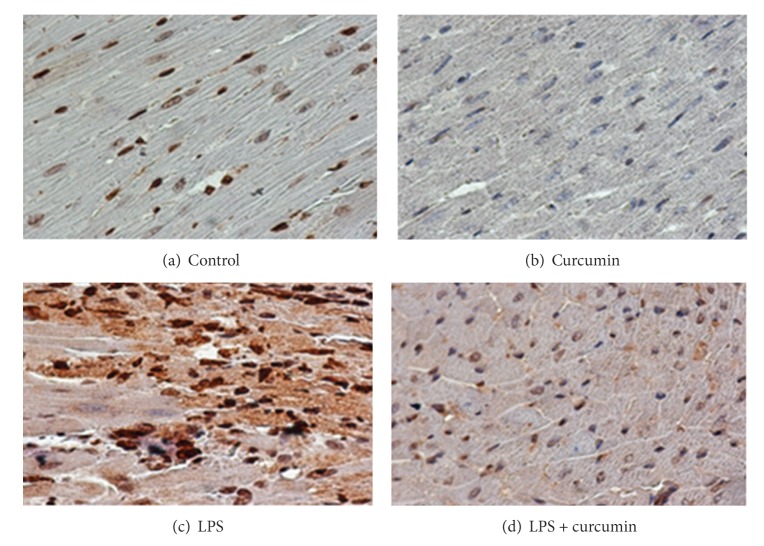
Curcumin attenuates histone H3 acetylation. LPS group showed that lipopolysaccharide (LPS) induced a significant increase in histone H3 acetylation, and curcumin group showed curcumin blocked LPS induced histone H3 acetylation.

In order to evaluate the mechanism of action of curcumin against LPS induced cardiac hypertrophy, we determined the condition of histone H3 acetylation by using acetylation specific antibodies. In this study, curcumin repressed the LPS induced acetylation of histone-3 in mice (Figure 4). As a control, the paraffin blocks were exposed to histone H3 antibody, and endogenous levels of histone H3 were detected by H3 antibody (Figure 3). As expected, LPS induced a significant increase in histone acetylation that was blocked and sustained by curcumin (Figure 4).

Histone proteins were detected by histone H3 antibody in control (Figure 3(a)), curcumin (Figure 3(b)), LPS (Figure 3(c)), and curcumin + LPS (Figure 3(d)) groups. Histone expression in nucleus was almost similar in all the conditions. But the cardiac cells in the LPS treated group showed more staining in the cytoplasm indicating hypertrophy. 

Figure 4 illustrates the degree of histone acetylation in cardiac tissue occurring in each treatment group. As can be seen in Figure 4(c), LPS induced a significant increase in histone acetylation as indicated by the intense staining. The histone proteins were mainly acetylated in the N-terminal end of the histone protein on lysine residues. In the curcumin + LPS treatment group (Figure 4(d)), there was a much lesser degree of nuclear staining with staining being similar to the control group, indicating that curcumin inhibited the histone acetylation activity of LPS. 

 These findings indicate that acetylation of histone tails by histone specific antibodies, mediated by histone acetyltransferases, is involved in activation of gene expression in the myocardium. Conversely, repression of gene expression can be induced by histone deacetylation, which is mediated by histone deacetylases (HDACs). Pretreatment with curcumin, which has a HAT inhibitory activity, attenuates histone modifications.

### 3.3. Curcumin Attenuates Histone H4 Acetylation

Modulation of chromatin structure has a central role in the regulation of transcription in eukaryotes [5]. In eukaryotes, histone H4 acetylation at the position of lysine 16 (H4-K16Ac) is a reversible posttranslational chromatin modification [14]. The incorporation of this modified histone into the nucleosome inhibits the formation of compact 30 fibers and inhibits the ability of chromatin to form cross-fiber interactions [14]. Acetylation of histone tails, mediated by histone acetyltransferases, is involved in activation of gene expression in the myocardium [5]. Conversely, repression of gene expression can be induced by histone deacetylation, which is mediated by histone deacetylases (HDACs). Pretreatment with curcumin, which has a HAT inhibitory activity, attenuates histone modifications [15].

Histone H4 acetylation at the position of lysine 16 (H4-K16Ac) is a posttranslational chromatin modification. In addition to histone H3 we also measured histone H4 acetylation by measuring the [^4^H] acetate incorporation into histones using H4 acetylation specific antibody. 

**Figure 5:**
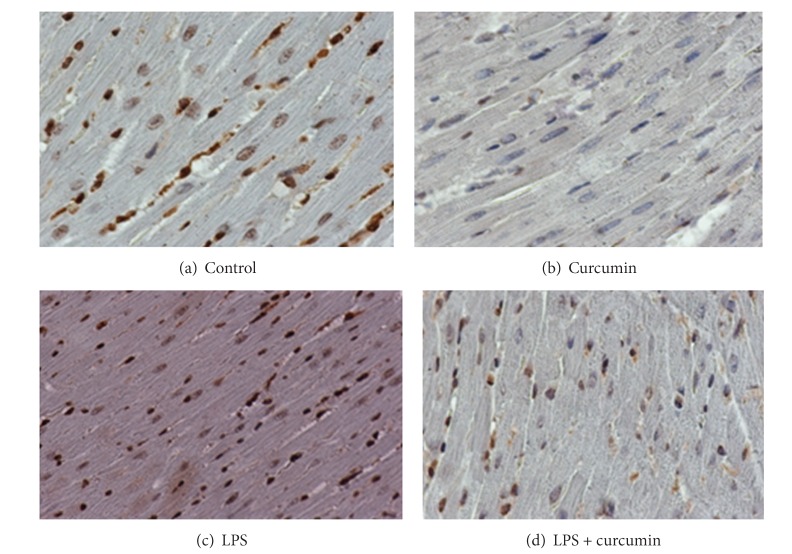
Histone proteins were detected by histone H4 antibody in control (a), curcumin (b), LPS (c), and curcumin + LPS (d) treated groups.

Figure 5 illustrates the level of histone proteins as detected by histone H4 antibody in cardiac tissue from control (Figure 5(a)), curcumin (Figure 5(b)), LPS (Figure 5(c)), and curcumin + LPS (Figure 5(d)) treatment groups. As can be seen in the figure, there was little variation in histone H4 basal expression levels among any of the treatment groups.

**Figure 6:**
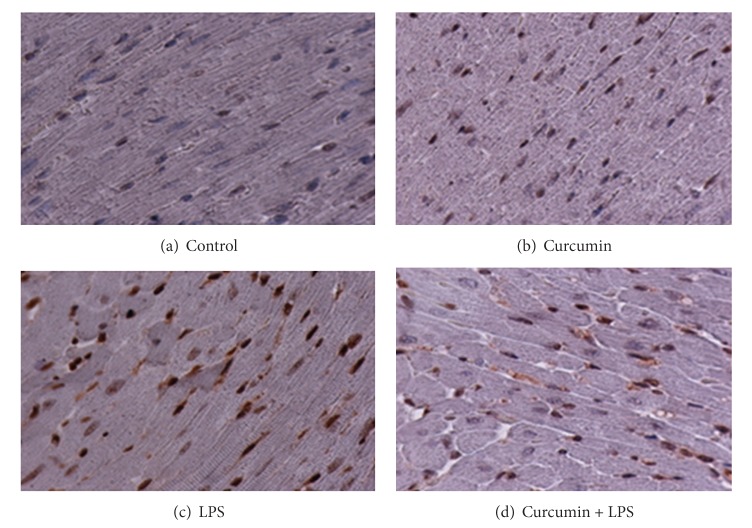
Curcumin attenuates histone H4 acetylation. LPS treated group (c) showed marked histone acetylation and curcumin + LPS treated group (d) showed curcumin reverses histone H4 acetylation.

The histone (H4) acetylation results are illustrated in Figure 6. As can be seen in Figure 6(c), the LPS treated group showed a significant increase in histone [^4^H] acetylation. Control and curcumin alone treatment groups, Figures 6(a) and 6(b), respectively, showed little difference in the staining of the acetylated histone H4. Similar to histone H3 acetylation, LPS induced H4 acetylation was also blocked by curcumin treatment (Figure 6(d)). This data strongly indicates that curcumin inhibits the acetylation of histones induced by LPS. 

Our findings also suggested that histone acetylation is associated with pathological cardiac hypertrophy. HATs (histone acetyl-transferase) acetylate histone proteins, relax chromatin, and expose genes for activation by cardiogenic transcription factors. Inhibition of histone acetylation has a central role for the antihypertrophy activity of curcumin.

### 3.4. Curcumin Attenuates LPS Induced p300 Acetylation

Two highly related and widely expressed molecules, cAMP regulated enhancer binding protein (CBP) and p300, have emerged as important cofactors for a broad number of transcription factors [16]. Both have histone acetyltransferase (HAT) activity and are important in gene transcription [16]. CBP was originally discovered based on its ability and to interact with the cAMP response element binding protein [17], whereas p300 was isolated as a cellular target of the adenoviral oncoprotein E1A [18]. Sequence analysis of CBP and p300 has shown substantial homology that these proteins are transcriptional coactivators [19].

p300, high molecular weight transcriptional factor, has three cysteine and histidine-rich regions. p300 knockout mice showed the defects of cardiac muscle differentiation and trabeculation, indicating the importance of p300 for early cardiac morphogenesis and heart development. However, p300 is also involved in the pathological process of cardiac hypertrophy [20]. Curcumin (diferuloylmethane), a major curcumanoid in the spice turmeric, is a specific inhibitor of the p300/CREB-binding protein (CBP) [21].

**Figure 7:**
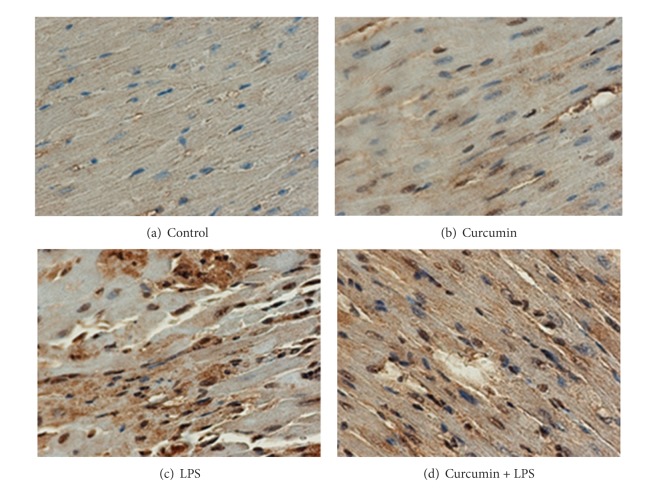
Curcumin attenuates p300 acetylation. LPS treated group (c) showed marked p300 acetylation, and curcumin + LPS treated group (d) showed curcumin reverses LPS induced p300 acetylation.

p300 levels were markedly increased by stimulation with lipopolysaccharide. To further check the mechanism of action of curcumin, we analyzed p300 protein acetylation levels. The results are illustrated in Figure 7. Curcumin did not affect the protein levels of p300. Curcumin inhibited the lipopolysaccharide-induced acetylation resulting in an increase in the binding of p300 to the gene promoter (Figure 7(c)). As was seen with histone acetylation, p300 acetylation was also inhibited by curcumin (Figure 7(d)).

p300 is involved in the pathological process of cardiac hypertrophy. The results of the present study indicate that inhibition of histone acetylation is a key mechanism for the anti-cardiac hypertrophy activity of curcumin and that p300-HAT serves as its molecular target. p300 may maintain basal function in the normal heart but it also promotes cardiac hypertrophy under LPS infusion. Our data also indicate that curcumin inhibits p300-HAT activity.

## 4. Conclusion

We can conclude from our study that curcumin attenuated LPS induced cardiac hypertrophy in vivo. Cardiac hypertrophy can be monitored by increased protein synthesis and induction of gene expression. Curcumin plays its anti-cardiac hypertrophy role by the involvement of chromatin remodeling, especially histone acetylation. Acetylation of lysine residues upon a single histone tail (hyperacetylation) would produce a stronger effect. Histone acetyltransferases have a critical role to acetylate histone proteins, relax chromatin, and expose cardiac hypertrophy genes for activation by cardiogenic transcription factors. Cardiac hypertrophy is also responsible for involvement of transcriptional factor p300. HAT activity in muscle is determined by p300 that causes modification of chromatin and associated transcription factors and gene expression. LPS induced cardiac hypertrophy accentuates p300 transcriptional activity, and LPS mediated cardiac hypertrophy is reversed by blocking of p300-HAT activity. Therefore, blocking p300-HAT activity may be a viable method for the treatment or prevention of myocardial hypertrophy.

The results showed that curcumin attenuated LPS induced cardiac hypertrophy in rodents and that the most probable mechanism of action involves inhibiting the p300-HAT activity.
